# Editorial: Corneal disease: an update

**DOI:** 10.3389/fmed.2023.1181810

**Published:** 2023-04-17

**Authors:** Luigi Capasso, Maddalena De Bernardo, Maria Borrelli

**Affiliations:** ^1^Banca degli Occhi della Regione Campania, PO dei Pellegrini, ASL Napoli 1 Centro, Naples, Italy; ^2^UOC Oculistica, Ospedale del Mare, ASL Napoli 1 Centro, Naples, Italy; ^3^Eye Unit, Department of Medicine, Surgery and Dentistry, “Scuola Medica Salernitana”, University of Salerno, Salerno, Italy; ^4^Department of Ophthalmology, University of Düsseldorf, Düsseldorf, Germany

**Keywords:** cornea, external disease, refractive surgery, keratoconjunctivitis, keratoconus

In this Research Topic, clinicians will find “food for thoughts” on the diagnosis and treatment of corneal pathologies throughout interesting original articles as well as case reports and reviews of the international literature.

In the article by Jan et al., the authors will focus on patients with Atopic keratoconjunctivitis (AKC), which often present itself in association with several anterior segment and eyelid diseases. The risk of recurrent corneal erosion, a relative common corneal condition, in patients with AKC will be analyzed in this study.

Corneal neovascularization, a sight-threatening condition, and the second cause of blindness worldwide, can be provoked by several ocular diseases, mostly associated with acute or persistent inflammation. The article by Lasagni Vitar et al. will try to measure the impact of corneal neovascularization on visual acuity and corneal sensitivity, providing an evaluation of its prevalence in patients affected by ocular surface diseases.

Keratoconus, an ectatic disorder with highly complex and varied causes, characterized by progressive steepening of paracentral cornea, with irregular astigmatism and visual function worsening, is the main topic of three articles of this Research Topic.

In particular, the study by Nicula et al. will focus on patients with abnormal findings in topography and tomography maps but with subclinical or clinical Keratoconus, in order to assess the efficacy of topographical and tomographical indices given by the Scheimpflug tomography.

The case report by Nuzzi et al. will discuss the management of a young keratoconus patient, initially treated with corneal cross-linking and subsequently implanted with intracorneal rings segment for visual rehabilitation. The patient, complaining visual acuity reduction and ocular discomfort, during slit lamp examination, presented superior ring extrusion, corneal thinning, and micro-perforation into the anterior chamber.

In the article by Ahn et al., authors will discuss the application of artificial intelligence for keratoconus screening, based on the results of basic ophthalmologic examinations.

Trichilemmal carcinoma (TLC) is a rare malignant adnexal tumor that usually develops as a large, solitary, multilobular lesion, providing a typical histopathology keratinization, and a peripheral palisading pattern. The article by Zhang et al. will show a case of corneal perforation caused by eyelid margin trichilemmal carcinoma.

Currently, corneal refractive surgery (CRS) is widely used to correct refractive errors. It is well-known that this procedure induces changes in the corneal structure and its biomechanics (1) which will make intraocular pressure (2), corneal power measurements (3), as well as intraocular lens power calculation (4) challenging. However, if CRS can induce anterior chamber depth and axial length changes (5, 6), is a still debated Research Topic, that will be discussed in the review “Unexpected ocular morphological changes after corneal refractive surgery.”

The review article by Ramachandran et al. will discuss the role of autophagy, a mechanism whereby cells respond to stress, and mitophagy, a selective form of autophagy, acting in clearing damaged mitochondria. Impairment or inhibition of these processes have been linked to corneal diseases, degenerations, and dystrophies. The review will summarize the current body of knowledge on these corneal processes, helping to better understand the mitochondrial dysfunction.

Overall, the eight articles in this Research Topic: *The cornea is not a piece of plastic: lesson from the last 10 years*, will provide a broad discussion of recent insights into corneal diseases that covers consolidated knowledge and interesting developments described in clinical studies as well as in basic science.

In the last 10 years the cornea has been incised, excised, heated, implanted, flapped, zapped, and cross linked, in the future can be plasticized also (7).

We believe the collections are informative for basic researchers, clinical scientists, and patients.

## Author contributions

LC, MD, and MB contributed to conception, design of the study, and wrote the manuscript. All authors contributed to manuscript revision, read, and approved the submitted version.
